# Strategies of prosociality: Comparing Nordic and Slavonic altruism toward Ukrainian refugees

**DOI:** 10.3389/fpsyg.2023.1065889

**Published:** 2023-03-08

**Authors:** Mads Larsen, Nina Witoszek

**Affiliations:** Centre for Development and the Environment, University of Oslo, Oslo, Norway

**Keywords:** anti-systemic altruism, authoritarianism, evolutionary psychology, migration, multilevel selection, qualitative interviews, systemic altruism, well-being

## Abstract

Nordic high-trust societies are underpinned by *prosociality*, a term denoting cooperation and working for the good of others. State-funded voluntarism provides opportunities for altruism that appears to contribute to the Nordics’ exceptional level of well-being. Altruists are rewarded by a warm, lasting affect that enhances personal well-being, thus motivating further prosociality. Humanity’s evolutionary past coded into us a desire to strengthen our community by helping those in need—a biocultural drive that is corrupted when authoritarian regimes enforce unselfish behavior on disempowered populations. Such *coercive altruism* has a line of adverse long-term consequences for communal functionality and individual flourishing. Our study examines how sociocultural context influences people’s prosocial strategies, and how sharing insights and practices from democratic and authoritarian traditions can lead to new, revitalized forms of altruism. Our in-depth interviews (*n* = 32) of Nordic and Slavonic helpers of Ukrainian refugees in Norway (1) illuminate the impact of culture and memory on altruistic practices, (2) define points of tension between systemic and anti-systemic modes of prosociality, and (3) identify cross-cultural interactions that generate trust, well-being, and social innovation. The post-communist experience of the Slavonic informants motivated *anti-systemic altruism*, which highlights spontaneity, improvisation, and occasional rule breaking. Norwegian *systemic altruism* is based on trust, efficacy, and rule-following. Our evolutionary approach to cultural psychology substantiates how important it is for development and immigration policies to align our knowledge of human nature with insights into the workings of cultural legacies. A better understanding of the biocultural mainsprings of altruism could be of crucial importance in our era of reemerging authoritarianism and increasing migration.

## 1. Introduction

The Nordic nations are often put forth as models for emulation (Meyer, 2007; Brandal et al., 2013; Andersen and Björkman, 2017; Helliwell et al., 2020). Income equality, gender equality, low-conflict politics, and prosperous economies with generous benefits contribute to social cohesion, as well as high subjective well-being, a measure of how people assess their own quality of life (Diener, 1984). According to several United Nations rankings, in the 21st century, the Nordics represent the pinnacle of human flourishing (Conceição et al., 2020; Helliwell et al., 2022). Nordic experts and politicians, impelled by their countries’ humanitarian mission, have presented their social democratic model as suitable for universal export (Tvedt, 2017). An evolutionary perspective on the emergence of Western modernity, however, reveals the importance of the cultural psychology that underpins many Nordic practices and institutions. The latter are difficult to transfer to nations with different social and cultural histories (Henrich, 2020). One such factor is high social trust (e.g., Rothstein, 2005; Trägårdh, 2013). Even a peremptory comparison between the Nordics and the Slavonic^1^ people is telling: among Norwegians and Swedes, 72 and 63%, respectively, think “most people can be trusted.” 23% of Russians, 24% of Poles, 30% of Ukrainians, and 40% of Belarusians think the same (WVS, 2020).^2^ The *World Happiness Report* ranks the five Nordic nations 1, 2, 3, 7, and 8, while Poland is 48, Belarus 65, Russia 80, and Ukraine 98 (Helliwell et al., 2022).

We posit that the evolutionary perspective is a fruitful tool for understanding differences in cultural psychology. Zagaria et al. (2020) proposed that using evolutionary psychology as an integrative theoretical framework could help the field of psychology enter a paradigmatic stage. Baucal and Krstić (2020) supported that researcher’s move in such a direction, but emphasized that the qualitative difference between biological and cultural evolution necessitates additional tools and frameworks. Our study aligns with this position. We ground our exploration in multilevel selection (MLS), an evolutionary framework that explains our species’ altruistic behavior toward non-kin (Wilson and Hessen, 2018). To understand how these universal lower mental functions express themselves in culturally mediated higher mental functions, we conduct a semiotic analysis to interpret how cultural narratives, tropes, and symbols inform our interviewees’ experience and meaning-making (Eco, 1986; Lotman, 1990). Our MLS model for well-being conceptualizes the universal, evolutionary foundation for altruistic motivation while also taking account of cultural histories that we share with others. Since cultural practices do not evolve like biological traits—that that is, through divergence mostly without amalgamation—using conventional explanatory tools from the evolutionary sciences has its limitations. In cultural evolution, transmission and cross-connections are common (Zagaria et al., 2021). A main purpose of our study is to investigate how cooperation between altruists who are influenced by distinct cultural psychologies provides fertile ground for cross-pollination and novel amalgamations of prosocial strategies.

Grounding our investigation in the universal mechanisms that drive altruistic behavior allows us to use an evolutionary argument against authoritarianism. It is not our intention to offer an in-depth, evolutionary approach to the relationship between authoritarian oppression and societies’ resilience and productivity. Rather, while acknowledging that authoritarian rule can be adaptive in some environments, we hypothesize that the authoritarian emphasis on coercive cooperation ultimately has an adverse effect on social trust and altruistic behavior, especially for populations that suffer economic and existential hardship. State oppression diminishes a group’s long-term efficacy through its misalignment with the basic drives of human nature in several regards (Welzel, 2013). Such governance also affects the human quest for meaning, which is the very fabric of culture (Baumeister, 2005). Religious beliefs activate our neurocognitive suite for meaning in ways that can synchronize and empower populations (Bellah, 2011; Harari, 2014). When modern ideologies fail to maintain such a hold on a moral community, the social fabric starts to unravel.

In the Nordic countries, there is a long tradition of socialization—that is, replication of positively charged narratives and practices—that have motivated voluntary, spontaneous prosociality. Such practices have been anchored in trust, individuality, and responsibility in a manner that has strengthened cooperative practices while enhancing well-being for the altruists (Witoszek and Midttun, 2018). This was often not the case in communist countries. Routinely, prosocial contributions were coercively extracted from subjugated populations by the ruling political elites. In the short run, this compulsory prosociality could promote group cohesion and efficacy—and even elements of well-being (Meier and Stutzer, 2008). Such prosociality, however, could not be sustained because it was undergirded by the deletion of personal individuality and responsibility, diffusion of mistrust, hatred of *the other*, and strong punitive measures imposed on outliers (Witoszek, 2019).

Communist rule has had adverse, long-term effects on cultural psychology in terms of disempowerment and distrust (Krastev and Holmes, 2020). In the cultural memory of citizens from former communist countries, work for the common good and contributions to the well-being of others are often associated with a loathed past, so that far more people eschew formal and informal voluntarism compared to in Western countries (Meier and Stutzer, 2008; Plagnol and Huppert, 2010). Unfreedom, ill-being, and low trust have created a legacy that is hard to shed off. Having been a part of the Soviet Bloc is a strong predictor for a population’s negative affect (Deaton, 2010). Miniscule levels of social trust, and associations of social cooperation with coercion and subjugation have contributed to how democracy in the former communist countries has suffered a backlash in the 21st century (Krastev and Holmes, 2020). Gaining a better understanding of the evolutionary and cultural mechanisms that promote or hinder democratic development has become pressing.

In this study, we compare the prosocial strategies of voluntary workers in Norway—native Nordic citizens and Slavonic immigrants—who help Ukrainian refugees. Our definition of voluntarism is the practice of working for the benefit of others without compulsion or promise of renumeration. We conduct in-dept interviews to explore the biocultural mainsprings of their modes of altruism. Our findings suggest that people whose prosocial habits were shaped, or rather distorted, by an authoritarian past, can reprogram themselves through actions that generate trust and self-confidence. We conceptualize the altruistic traditions from Nordic democracies and Slavonic authoritarianism as, respectively, *systemic* and *anti-systemic altruism*. Our comparison of culturally-informed strategies of prosociality has a threefold aim: (1) to draw attention to the importance of cultural history and memory in the habits of the heart and mind of culturally heterogeneous groups of volunteers; (2) to explore interactions between prosocial groups as a locus of tensions and a stage of mutual learning that can inspire more culturally sensitive forms of prosociality; and (3) to show how prosociality affects personal transformation and social innovation through cross-cultural interactions.

## 2. Theoretical framework

Since Diener (1984) popularized well-being studies among psychologists, the field has become among the hottest topics of social science (De Vos, 2012). Positive psychologists have influenced politicians to try to move away from overly focusing on GDP and other economic metrics. In 2011, the United Nations unanimously adopted the resolution “Happiness: toward a holistic approach to development.” Since then, several dozen countries have adopted national well-being accounts (Diener et al., 2015). Positive psychology’s Western-centric approaches (Uchida et al., 2009; Uchida and Kitayama, 2009; Rappleye et al., 2020; Krys et al., 2021a,b), in addition to its conceptual overabundance (Røysamb and Nes, 2016), have hindered the development of a credible cross-cultural model for well-being. Calls for an evolutionary approach have mostly been disregarded (Buss, 2000; Nesse, 2005; Hill and Buss, 2008). We have responded to these calls by bringing together insights from the well-being field under an umbrella of multilevel selection (Larsen et al., 2023).

The current third wave of evolutionary thought highlights MLS as a framework that explains human altruism (Wilson and Hessen, 2018). Competition and selfishness may provide adaptive advantages for individuals within a group, but since groups of effective collaborators tend to outcompete groups of selfish individualists, intergroup warfare and competition have provided evolutionary pressures that promote prosocial practices.^3^ Culture can work counter to our selfish instincts by extending our natural predispositions for nurture to non-kin individuals or even strangers (Lumsden and Wilson, 2005; Wilson et al., 2014). Internalized cultural ideals thus facilitate effective prosociality by turning extrinsic group motivation into intrinsic behavior of members of the group. By acting in ways that one’s culture defines as meaningful, individuals are rewarded by an increase in self-esteem, an important component of well-being (Solomon et al., 2000; Kirkpatrick and Navarrete, 2010).

An MLS perspective brings attention to how individuals are torn between working for their own success and that of their group. Solving adaptively relevant problems for themselves and their close kin or social group provides a good feeling that promotes such behavior. Yet sacrificing for the benefit of others, or the abstract ideals of one’s group, can engender even more intense and lasting affect for the altruist (Baumeister, 2005; Baumeister et al., 2013). Well-being societies, we posit, are those that effectively facilitate multilevel well-being, that is, they nourish individual flourishing through a balance of competition, cooperation, and widespread opportunities for altruism. We use this MLS perspective to offer an evolutionary model for what makes humans conclude that they have a good life: Happiness + Meaning = Well-being.^4^ Both happiness and meaning are biocultural phenomena, affects that have a biological foundation, yet are strongly mediated by culture.

Our model conceptualizes happiness not as an affect meant to be maximized, but a semi-transient reward for solving adaptively relevant problems. Money, status, social and professional success, and many other achievements, enhance an individual’s ability to survive and reproduce. When we progress toward such goals, positive emotions signal that we should keep on acting in the same way (Nesse, 2005; Hill and Buss, 2008). It is the progression toward these goals, rather than the goal achievement itself, that is the imperative (Carver and Scheier, 1990). Indirect fitness concerns, as well as the adaptive benefit of sociality (Lewis et al., 2015), let us reap happiness from spending time with our immediate circles and also appreciating their successes. Baumeister’s work on meaning demonstrates how people are affectively rewarded for contributing to their community (Baumeister, 2005; Baumeister et al., 2013). Pursuing a sense of meaning is adaptive since this affect assesses, and reinforces, social belonging. For purposes of analysis and policy recommendation, we posit that it can be profitable to conceptualize well-being to consist of these two clusters of affects, which promote individual and group selection, respectively.

This evolutionary model is culturally neutral in the sense that all communities consist of people who face often conflicting pressures related to individual and communal needs. Such a perspective frees us from the ontological and semantic discussions around well-being that inevitably become culturally biased. Long-running debates have focused on what well-being should be or which affects—or virtues—it consists of. Millennia of Western philosophizing on well-being and altruism impose value-charged assumptions on people’s thinking that culture makes invisible (Ricard, 2015; Wilson and Coan, 2021). Striving for cultural objectivity, for instance through a model grounded in the humanistic values of human rights (Vittersø, n.d.), overlooks the Western origins of such individual-centered values (Finnis, 2011). Some Confucian perspectives go against Western insistence on well-being having to be assessed on the individual level. Confucians stress interdependent well-being—the importance of good relationships and social harmony—against which individual happiness can be perceived as a threat (Krys et al., 2021b). Our MLS model can accommodate this conflict. Whether independent or interdependent happiness pursuits are more adaptive, depends on the sociocultural context. In kinship societies, the well-being of one’s kin group is of such importance to each individual’s fitness that interdependent concerns take precedence (Henrich, 2020). In Western societies, individual strategies are paramount.

While our model’s cultural neutrality applies to its overarching MLS framework, understanding how distinct cultures motivate certain behaviors in regard to happiness and meaning requires careful historical and cultural research. Insights gained within one cultural sphere must not be applicable in another (Baumeister, 2005; Heine, 2010; Baucal and Krstić, 2020). Our findings within one Nordic country are therefore tentative in terms of their cross-cultural applicability. To comprehend well-being itself, however, it suffices—within our model—to view our well-being system as a biocultural phenomenon that makes people “feel good” in a manner that motivates them to continue the behavior that triggered this affect.^5^ Precisely which feelings these are or what they motivate will vary with culture, as will terminology. Since *happiness* and *meaning* are used in popular and scholarly discourses for a variety of purposes,^6^ abbreviating these terms to “H” and “M” would be an option, yet we prefer to use familiar words for ease of communication (Larsen et al., 2023).

Applying a model of purported cultural neutrality to argue against authoritarianism could appear as another expression of Western-centric ideology (Kemmelmeier et al., 2003). We therefore stress that our analysis in this regard limits itself to one mechanism: the undermining of long-term trust and well-being as a consequence of coercive altruism. Whether such a downside could be worth the upside—from an evolutionary perspective—depends on context. The primary purpose of that part of our analysis is to offer a partial explanation for why it has been so challenging to forge thriving democratic institutions in nations with an authoritarian past.

## 3. Materials and methods

Positive psychology has relied almost exclusively on quantitative research. If well-being is not best understood as a consequence of life factors, but from how individuals interpret and adapt to evolutionarily evolved signals, states Nesse (2005), “survey studies of well-being will overlook most of what is important.” He concludes that “implications for methodology are severe [as] only narrative includes information detailed and idiographic enough to allow a real understanding of an individual’s life.” This insight informs our qualitative approach which combines in-depth semi-structured interviews, narrative analysis, and our previous work on the cultural history of the Nordic and Slavonic regions (e.g., Witoszek, 2007, 2011, 2019; Witoszek and Midttun, 2018; Larsen, 2021, 2022).

### 3.1. Study population and design

To investigate the mainsprings of prosocial behavior, we recruited 32 dedicated altruists—people who had committed to helping refugees without payment—in order to gain access to thick descriptions of altruistic motivation. We contacted local leaders in the Red Cross who forwarded our request to members. We found informants through social media groups dedicated to help Ukrainian refugees, and a few were recruited after appearing in news media. Our project was presented as investigating the relationship between altruism and one’s personal well-being. Naturally, our selection is not representative. For an initial study of the MLS mechanisms of prosociality, our priority was through our purposive sampling to gain info-rich access to the narratives of people who had considerable experience with and reflection around altruistic work. A few had only had limited interaction with Ukrainians so far, but still a background of helping refugees from a variety of nations.

We recruited 16 females and 16 males in the age range 23–80. Nearly all were long-term residents of Norway. Fourteen identified as primarily Norwegian, one as Swedish, 10 as Polish, four as Ukrainian, one as Russian, one as Belarusian, and one as Latvian (Table 1). We were a group of scholars who conducted interviews in Polish, English, Norwegian, and Norwegian-Swedish. Interviews in Polish were transcribed manually—the other ones *via* software, then quality-proofed manually. Direct quotes are edited for readability. We use this research material in another article that elaborates on our well-being model (Larsen et al., 2023).

Our grounded theory approach entailed an interplay between data collection and analysis throughout the interview period March–June, 2022. Twenty interviews were in person, while 12 were *via* Zoom due to these informants’ remote location. Informed consent was obtained from the informants for the publication of any potentially identifiable images or data included in this article. Only a few selected to be anonymized in terms of full name, yet we choose to describe informants using no more than nationality, gender, and age. With a relatively large sample size for a qualitative study of this type (Marshall et al., 2013; Schreier, 2018), we present informants with such low level of detail that names are less relevant. We respected the request of female informants of Polish extraction who preferred not to disclose their age and proposed that we instead use their first names. We stopped recruiting when reaching saturation in terms of novel information per interview. Ethics approval was obtained in line with the Norwegian decentralized model. Our project was assessed by the Norwegian Agency for Shared Services in Education and Research (reference number 445357).

**Table 1:** Interviewees.

Nationality	Sex	Age or name
Belarusian	Male	40–45
Latvian	Male	35–40
Norwegian	Female	35–40
Norwegian	Female	45–50
Norwegian	Female	60–65
Norwegian	Female	75–80
Norwegian	Male	20–25
Norwegian	Male	30–35
Norwegian	Male	35–40
Norwegian	Male	50–55
Norwegian	Male	60–65
Norwegian	Male	65–70
Norwegian	Male	65–70
Norwegian	Male	65–70
Norwegian	Male	70–75
Norwegian	Male	80–85
Polish	Female	Agata
Polish	Female	Aleksandra
Polish	Female	Anita
Polish	Female	Ewa
Polish	Female	Hanna
Polish	Female	Marta
Polish	Female	Anon.
Polish	Female	Anon.
Polish	Male	35–40
Polish	Male	50–55
Russian	Female	35–40
Swedish	Male	30–35
Ukrainian	Female	30–35
Ukrainian	Female	35–40
Ukrainian	Female	40–45
Ukrainian	Male	35–40

### 3.2. Cultural history and narrative analysis

The Nordic countries have centuries of positive experience with bottom-up collaboration across social spheres. In the sixteenth century, choosing Lutheranism as their Protestant creed was the pivotal decision that set Nordics on a path different than those of other Europeans (Fukuyama, 2014). The dominant prosocial, egalitarian ethos made it everyone’s responsibility to ensure everyone else’s well-being, as all citizens—from pauper to king—were meant to be united in a “priesthood of believers.” The Nordic Model can thus be understood as a secularized version of Lutheranism that would be unlikely to work as effectively in nations with a different cultural past (Nelson, 2017). After WWII, Lutheran inclusivity was expanded through generous foreign aid and, later, hospitality to immigrants and respect for their traditions. After the 2015 migrant crisis, immigration policies became more restrictive and focused on integration (Beck et al., 2017).

The Slavonic region was less influenced by those practices that Henrich (2020) identifies to have driven WEIRD psychology (Western, Educated, Industrialized, Rich, and Democratic).^7^ These countries have a history of centralized, authoritarian rule with widespread serfdom and oppression that deprived individuals of agency and submitted them to the group. While decentralized Nordic communities often came together voluntarily to help each other during times of crisis, the Slavonic experience was often one of imposed labor and non-voluntary sacrifice. Further, the ideologically informed tradition of distrust casts a long shadow on the present (Kornai et al., 2004; Kornai, 2021). While Nordic citizens are exceptionally trusting of their governments, the dominant Slavonic attitude to institutions is one of opposition and subversion. The communist legacy of enforced, state-controlled altruism complicates the expansion of altruistic circles. Unlike Nordic citizens, most Slavonic people have adapted to new challenges through prosocial strategies that are interpersonal, inventive, and subversive toward reigning ideology.

These distinct pasts help us understand differences in the narratives and practices of prosociality from our culturally diverse informants in Norway, a country with one of the world’s highest rates of volunteerism (Huppert et al., 2009; Plagnol and Huppert, 2010). Our informants’ stories offer insights into how cultural memory influences altruistic practices and how these are infused with meaning. In terms of qualitative psychological research, our approach is evocative of the constructivist-interpretive tradition (Levitt et al., 2017), although our evolutionary perspective provides a meta-narrative with different implications than the constructivist one.

## 4. Results

A year after Russia’s attack on February 24, 2022, more than 8 million Ukrainian refugees have migrated to European nations (UNHCR, 2023). Around 40,000 came to Norway (UDI, 2023). The national refugee reception system was not dimensioned for the quantity of the influx. Especially in the first months, large unmet needs were partially covered by volunteers. The media attention and proximity of the conflict motivated unusually many persons to donate their time and resources, many of whom helped refugees for the first time. They offered a variety of services, from sharing information about the registration process and distributing practical objects like toothpaste and underwear, to comforting distressed refugees and offering transportation from the Ukrainian border to Norway.

That these refugees were victims of a relatively near-by military invasion that could spread to other European nations, affected the volunteers’ motivation and response. Our Slavonic informants reported being particularly distressed by a war that reminded them of past traumas under Russian and Soviet oppression. For many of our Nordic informants, too, helping Ukrainians felt personal due to a cultural and geographical proximity. Many had helped Middle Eastern or African refugees in the past, but without experiencing the same connection to the conflict that the refugees had escaped. Gentile (2020) substantiated how the Russo-Ukrainian war is less experienced by those afflicted as originating from an ethno-national division. How people self-identify as belonging to the Western or Russian cultural spheres is a stronger predictor for their sympathies. The war in Ukraine is by many perceived as a conflict of distinct civilizations that has an impact on the future of Eastern European borderlands (Gentile, 2022). In the West, the Russian threat has motivated a stronger sense of European identity, but there is a marked difference is European societies’ response to the war, depending on their proximity to Russia and the size of their Russian minority population (Gehring, 2022). This differentiation in response also contributed to how distinct cultural psychologies motivated different prosocial strategies toward Ukrainian refugees.

### 4.1. Nordic systemic altruism

In the post-WWII period, Norwegian altruism was institutionalized in a “humanitarian-political complex” (Tvedt, 2017). Generous aid—and later, mass-scale immigration to Scandinavia—were meant to expand social democratic practices to cultural others (Skagen, 2018). This generosity was underpinned by Scandocentric universalism, the assumption that the egalitarian, conformist Nordic Model was more “civilized” and humane than other social orders, and that most people would embrace Nordic values and practices once properly exposed to these. This ethos—a gentle, Nordic version of the West’s end-of-history hubris (Fukuyama, 1992)—has in many regards been weakened in our present era. Native critics have deconstructed narcissistic assumptions (Tvedt, 2017; Norman, 2018; de Puyvallée and Bjørkdahl, 2021), and the past decades have demonstrated that not all the world’s people desire to live in liberal democracies. Nordic universalism, however—and the institutionalization of altruism—still inform the region’s narratives of prosociality that underpin how they welcome refugees. Most of our Nordic informants had internalized that altruistic activities should (1) be highly organized through state-funded institutions like the Red Cross, (2) provide fair, uniform services for all types of refugees, and (3) prioritize long-term efficacy over short-term emotional reward.

While providing such rational, knowledge-based altruism rarely offered peaks of high emotional reward for our Nordic interviewees, they all experienced an increase in long-term well-being from engaging in what they perceived to be meaningful activities. “I have always had the feeling that I wanted to help others,” said a Norwegian male (30–35), “I get self-confidence from contributing to other people doing well.” He would drive an hour each way to volunteer at a center that expedites Ukrainian refugees on arrival. His tasks did not include socializing, but to organize a storage room and keep watch: “It felt like I was just waiting for 10 hours, but as long as I feel tired afterward, I feel useful.” Another Norwegian (20–25) got a good feeling from being part of a community of likeminded people: “I do not get the best feeling when I am talking with those we help, but afterward, when we as a team have succeeded with arranging an event to their benefit, I feel good.” A Norwegian female (70–75) seldom derived short-term satisfaction, which she referred to as “happiness,” from her weekly meeting with the refugee with whom she had been assigned to socialize. Being with her own friends or doing hobbies were more rewarding. Yet sacrificing for the benefit of others provided her life with a sense of meaning that sustained her well-being in the long run.

The majority of our Nordic informants chose to make altruistic contributions that aligned with this low-intensity ethos—although several felt the draw of social engagement that triggered stronger emotions. “We have been indoctrinated into this thinking, that if you want to change society, you must plan—you cannot just do what feels good. Getting state funding is important, and applying can take years. This weekend, we actually did something more action-oriented, we painted public benches in rainbow colors,” said our Norwegian informant (30–35), then self-effacingly whispered, “but we had applied for permission first.” He had volunteered much throughout his life, but always “wanted to know the frame first, the details, before I engage. What we do must be based on knowledge.” A Norwegian (35–40) was transformed by his experience with helping Ukrainians closer to the war zone, an activity that provided an intense reward, “It is difficult to focus on your own trivial problems when you have been in a situation like that, and it can become very difficult if not impossible to go back to the standard Norwegian consulting trade, and sit there and solve problems that do not feel as meaningful. So that is one of the reasons why I’m looking at possibilities to tweak my career in the direction of starting Ukrainian IT companies or help Ukrainian IT startups get contacts and clients.”

### 4.2. Slavonic anti-systemic altruism

Most of our Slavonic informants had lived for several years in Norway, but few had previous experience with voluntarism. They suffered from high levels of emotional turmoil after Russia’s invasion of Ukraine and wanted to help those directly affected by the war. Having grown up in nations that had a strong memory of first Russian and then Soviet oppression made their engagement with refugees more personal. For some, intense identification with the war victims was a source of short-term emotional stress—much higher than what most Nordic helpers experienced—but the altruistic activities infused their lives with a sense of meaning that had significant long-term benefits. The Slavonic volunteers felt empowered, expanded their social networks, and several experienced personal transformations. A Ukrainian female (30–35) felt that she was getting a new life, that she learned to make difficult decision and solve problems. A Polish volunteer, Hanna, said,

I am not an activist, so before I started helping refugees, I went through the classic phases: first skepticism, then amazement, because I did a lot more than I planned, then the excitement of working with others and realizing that I’m changing someone’s life for the better, and finally the feeling of creating my own better self in a previously empty space. In the local community, I’m no longer Hanna the wife of the Polish doctor, a mother of three children, and a good wife. I’m Hanna the organizer, a public person. My well-being is related to family for sure, but there are these peaks that only extra-family functions can give you.

Another Pole, Anita, experienced a similar epiphany. She had read that the most enduring sources of well-being were connected to one’s family, “but I discovered that the top of the pyramid is the work for the others. I developed skills I did not think I had, like being social, flexibility, and the ability to get out of my role as the mother who sacrifices everything on the family altar.” Several Slavonic informants were surprised at how much well-being they derived from helping Ukrainian refugees. Ewa said that since she started volunteering at the refugee center, “I sleep better, I do it for egotistic reasons, for my own peace of mind, I’m not Mother Theresa. But then, when I hug a person who is in distress, and cry with her, I have a sense that I mean something in the cosmos.”

Coming from nations in which spontaneous prosocial activities had been disincentivized, the Slavonic informants had to learn voluntarism as they went along. A Belarusian male (40–45) explained that in his native culture voluntarism was typically done by people with selfish political interest. A Ukrainian male (35–40) emphasized that where he came from people hardly trusted any institutions or their representatives. A Ukrainian female (35–40) said, “We cannot trust institutions to help us, and people make malicious gossip, so it is better not to get involved.” Informed by such a culture of fear and mistrust, some Ukrainian refuges were initially wary of their helpers. A Norwegian female (45–50) said, “There is corruption up in the Ukrainian higher systems, so they are not used to that culture of Norwegians very often acting selflessly. I have always been a person who feel that I can help without needing to get anything back for it. I get so much back just from the time we spend together, really just to feel that I have helped someone.” Gaining insight into the rewards individuals often experience when helping others, several Slavonic informants bemoaned how their former sociocultural context had deprived them of such opportunities.

### 4.3. The consequences of coercive altruism

Meier and Stutzer (2008) documented how East German well-being decreased after the Cold War—partially as a result of reduced voluntarism. In this case, altruistic activity under the communist regime had been encouraged through societal mass organizations and other civic engagement groups that practiced an ethos of discipline and obedience to authorities (Gensicke, 2000). Longitudinal survey studies show how even partially coercive altruism had, on average, a beneficial effect on the altruists’ quality of life. Our evolutionary past has coded into our well-being system so strong rewards for helping others that altruism can elevate our mood even if we despise those who forced us to be prosocial against our will. After the German reunification, most people chose not to participate in remaining idealistic organizations, even if abstaining from altruist activities affected negatively their life satisfaction (Meier and Stutzer, 2008).

Plagnol and Huppert (2010) investigated why the volunteering rate has remained so low across the former Soviet Bloc. Social, psychological, or cultural factors related to volunteering do not explain why there is a tenfold variation across Europe—from 7% of Bulgarians to 67% of Norwegians reporting to have done voluntary work in the past 12 months. In the early 2000s, Poland, Russia, and Ukraine’s percentages were in the mid-10s to low 20s (Huppert et al., 2009). A possible main driver of this variation could be the fact that richer populations can afford to volunteer more of their time, but there is only a weak correlation between productivity and voluntarism (Kakoli and Ziemek, 2000). Plagnol and Huppert concluded that a country’s level of volunteering to a large extent is determined by its historical background and institutions. Kuti (2004) attributed Eastern European aversion to voluntarism to their associations of prosociality with the oppressive ideology of Soviet states. Communist parties had demanded that people sacrifice time and resources for social and political causes with which they did not necessarily identify. Since such activities had been tied to communism, the “concept of volunteering became obsolete” with the demise of communist regimes.

Our Slavonic informants were mostly too young to have experienced coercive altruism, but spoke of a strong pull from their countries’ communist past. Ewa explained:

The Poles are still haunted by the post-communist legacy, which is about a zero trust in the state and state institutions. This means that we are anti-systemic, we prefer to act outside the system, we feel safer in relating to a person more than to an institution. The Norwegians have a sense of security, they trust their government and institutions. They have this strong belief that eventually things will sort themselves out. The Poles do not have this belief. We cannot wait, because we literally feel the refugees’ fear of the unknown. So, the Polish style is: first help, and then formalities. The Norwegian style is the opposite: first formalities, and then help.

Not having an entrenched history of organized voluntarism to draw on, our Slavonic informants relied on a largely emotional response to the Ukrainian refugee crisis and on cultural impulses that had been coded into them before they immigrated to Norway. Since our shared nature rewards altruistic behavior also in disincentivizing environments, people seek outlet for their impulse to help. In former communist societies, especially Poland, a tradition anchored in contempt for, and insubordination to, state institutions informed prosocial strategies that highlighted subversion, interpersonal ties, and inventiveness. Several of our Slavonic informants first contacted established organizations in Norway, but found their bureaucratized volunteerism to misalign with their own cultural and emotional expectations. Many therefore decided to invent their own forms of *ad-hoc* voluntarism to meet their own needs and those of Ukrainian refugees.

### 4.4. The pull of anti-systemic altruism

The Nordic tradition goes against our species’ emotional impulses in the sense that rational, bureaucratized voluntarism may feel heartless unless you have been socialized into an ethos that makes sense of such practices. More often, in our evolutionary past, helping non-kin was facilitated through informal, interpersonal connections that our emotions reward. Several of our informants emphasized that they got the best feelings from face-to-face altruism. Directly aiding someone in dire need could feel euphoric, said a Norwegian male (50–55). This greater affective reward was one element that influenced the unorthodox ways in which many Slavonic informants organized their voluntarism. By foregoing organizational structures, instead contacting refugees directly and providing for their needs, helpers did what to them felt most natural and emotionally rewarding. The experience was so overwhelming that nearly all Slavonic informants overworked themselves to the detriment of their families or professional obligations. Such a mode of operation provided strong affect, but none believed that they could maintain this high level of activity for more than a few months. A Russian female (35–40) said of their efforts, “We only trust ourselves. We work hard, burn out, and quit. There is so much arguing. We also distribute unfairly what we collect for refugees.”

Some of our non-Norwegian informants had the impulse to challenge Norwegian routines—in spite of admiring their new country. A few stressed that they “really like Norway,” that the Nordic way of life strongly aligned with their preferences. Still, it felt wrong from them to resort to technocratic rationality when people fleeing from war needed emotional support. Many felt an aversion against the cooperation between volunteer organizations and private refugee centers—in spite of both types of organizations being funded by the Norwegian state. Idealism and business should not mix—it is an “unholy alliance,” as Ewa put it. She explained that the employees at reception centers “have a job. We have a mission. The two things collide, full stop.” Assigning themselves a status as idealists—in opposition to professional refugee helpers—seemed, for some, to fuel a sense of superiority in terms of their own morals and insights. This psychological mechanism helped justify their often-nonchalant attitude to Norwegian rules. A Russian (35–40) said, “They have so much trust in the system and assume everything will work out, but the system will need 2–3 months to catch up with what is happening now. The big organizations are not as adaptable as we can be, so we can fill many gaps for refugees now.”

To meet these needs, many Slavonic informants found likeminded people online and formed *ad-hoc* groups. Imaginative use of social media helped them organize quickly, gather requests from refugees, collect needed objects, and distribute these across the country. Friction could be considerable when interacting with different levels of state and local bureaucracy, established organizations, functionaries at refugee centers, and other formal and informal actors. Anita was part of a group that went to a hotel where refugees were living to discover that—

most refugees had spent days and weeks in the same underwear, in the same clothes. There was an epidemic of Covid among children, you name it. We wrote down what was needed, including a wheelchair for a disabled child. But the Norwegian functionaries disagreed. “You have to have a doctor’s referral for a chair,” they said, “then write an application, then wait and get a permission, and then we’ll discuss it.”

The Poles protested vehemently: “This mother and child cannot wait any longer, we need to get them out of here now.” In this case, the Norwegian volunteers sided with the Poles. Irritated by excessive red-taping, they proclaimed that “first we organize help, and then we can have a discussion.” One informant told us,

There was a sense of urgency, shared both by the Polish and Norwegian volunteers, so both started making phone calls and organizing transport, and they obtained a wheelchair on the very same day. We also smuggled groceries and gas stoves to the hotel rooms, so the Ukrainians could cook their own food and not eat the super-spicy Thai or Indian food that the Norwegian authorities serve to refugees from the Middle East.

Hanna discovered that, while the humanitarian system suffered from inertia, individual Norwegians could be moved more easily,

When I rang the Red Cross, they told me that in Norway refugee help was an institutional matter so they would not assist an ad-hoc group like ours. But then, I just went to neighborhood shops who all said yes to our request to put out special baskets for collecting food and medicines for refugees.

Such small victories made the Slavonic mode of anti-systemic altruism appear attractive to Norwegian volunteers who previously had not challenged how things were done. These groups joined forces and forged new strategies of prosociality that combined the Nordic methodical and systematic approach with Slavonic inventiveness. This transcultural, mildly rebellious prosocial community invigorated both parties and provided help that was more tailored to the needs of Ukrainian refugees. The affective rewards were considerable. Polish Agata said,

In the process of overcoming obstacles, you meet so many fantastic, totally “un-Norwegian Norwegians” you did not know existed. These are Norwegians who are not governmentalized (*statliggjort*)—they are anarchists and rebels. When we work together, for example, on organizing information for the refugees, or smuggling stuff to the refugee hotel, it feels like being part of a conspiracy, of the good guys united against the bad guys. There’s nothing to beat the feeling of giving refugees their dignity.

### 4.5. Cultural navigators with legs in both worlds

Our interviews point to the Slavonic immigrants in Norway functioning as a bridge between the Scandocentric universalism of the refugee system and the particular needs of Ukrainian refugees. Knowing both cultures, the Slavonic helpers could effectively challenge Norwegian assumptions and practices. Polish Aleksandra said,

The refugees are haunted by hundreds of questions. What’s going to happen to them? How long will they stay in a hotel or reception center? What are their rights? How can they learn Norwegian, and so on? We know something about a lack of information because we were born in communist Poland where a lack of information and misinformation were chronic. We knew that this can easily lead to a mental breakdown.

She conceived of the project *Ukrainfo*, for which five mothers worked around the clock to make a website with information for refugees in five languages. The site explains important cultural differences between Norway and Ukraine, and answers the most common questions that refugees had asked the volunteers. “After fixing the website,” Aleksandra said, “we felt proud as peacocks. Then came the anticlimax. We sent emails about this site to the Norwegian police, immigration authorities, you name it. No one replied. But we know that some municipalities use our information.”

Spurred on by such small successes, the same informant organized the collection of emergency contraceptive pills for victims of rape. Again, she had no time for Norwegian formalities,

If you compare our ways of collecting these pills with the actions of the Norwegian Humanist Association, you see the difference. The Humanist Association almost gave up when they found out how complicated it was. One had to send an application to the authorities for permission to get the pills, then provide instructions in the native language, then one needed somebody to control it and approve it, and so on. We went around these complications and managed to get 400 pills in a week.

In spite of the burden of Norwegian formalities, as the Slavonic immigrants gained experience as volunteers, many found that their trust in institutions and the Nordic mode of altruism increased. A Russian (35–40) self-effacingly told that their *ad-hoc* organization would soon be acquiring “an organization number” and writing their own “articles of association” (*vedtekter*). She annunciated the Norwegian terms in a way that made her and the interviewer chuckle. Months earlier, such bureaucratism had been anathema to her prosociality. Now being an effective part of a web of organizations that cooperated to the benefit of refugees, she had come to understand how being a fluid, spontaneous group of private citizens did not promote long-term efficacy. The Belarusian (40–45) who had begun our interview complaining about Nordic naivety and ineffective organizations, wanted Slavonic nations to learn from his Nordic experience. “It feels bad to be selfish,” he said, “A good future requires that people cooperate. Since we do not organize ourselves, we are easy to manipulate. I choose to be optimistic about the future, therefore it is okay to risk being naïve.” If he were to advocate for more voluntarism in Slavonic cultures, he would, for strategic reasons, emphasize Norwegian efficiency and productivity, “Look to Norway, I would say. We can get more prosperity if we can develop trust through cooperation. This makes for good culture with better communication and less crime.” A Ukrainian (35–40) said, “The best way to build trust is through volunteering and doing things together.”

Our interviews uncovered how cross-cultural cooperation resulted in several small, but appreciated changes at those refugee centers where Nordic and Slavonic volunteers worked together. A Polish male said (35–40),

In Norway, there is a system which may seem de-humanized, at least in the beginning. It was originally based on helping refugees from the Middle East. This system was projected into humanitarian help for the Ukrainians without taking into account the Ukrainian cultural values which are more European.

Slavonic volunteers convinced center employees not to refer to refugees with numbers only, but with their names. Part of the de-bureaucratization was to offer older refugees interpersonal help instead of iPads for purposes of registration. Food was made less spicy. These were not universal changes imposed top-down, but local adaptations driven through by volunteers who had legs in both cultural camps and could advocate effectively for customization. “Norwegians create this superior image of themselves as the chosen people and then fall in love with it,” said Aleksandra, “Everything they say and do with regard to refugees says: you are now in a splendid, civilized country and you’d better learn how we do things here. Then they quickly discover that many Ukrainians were educated, hardworking people, so they change their tune and became more respectful.”

A Swedish volunteer (30–35) noticed this change, but attributed it to how refugees now consisted of “blond women instead of brown men.” He and other Nordic informants experienced how working with refugees made them more aware of the shortcomings of their own culture. In the Middle East,

They have a more collective responsibility for each other. We Scandinavians have rationalized away our humanity. Child care, elder care, psychological care—the welfare state provides for everyone. We have lost what can give our lives meaning, so we drink beer on the weekends and look for meaning in all the wrong places. If all Scandinavians volunteered, everything would be better. The Nordic Model has given us many positive things, but we have lost much too. Something very important for humans is to treat other humans humanely.

## 5. Discussion

This article’s MLS perspective on altruism brings attention to how our evolutionary past programmed into us a drive to contribute to the well-being of others. The experiences of our Slavonic helpers illuminate how our universal prosocial dispositions are strongly mediated by cultural values and norms, political systems, and ideological creeds. Our informants exemplify how an authoritarian past can have adverse effects on development and modes of prosociality. In post-communist nations, coercive altruism had discredited voluntarism by tying prosociality to a despised, outdated regime. Generations later, it still felt strange, at least initially, for many Slavonic informants to invest in the well-being of strangers. While not all personality types benefit similarly from making altruistic contributions (Meier and Stutzer, 2008), for populations as a whole, widespread formal and informal voluntarism is a win-win proposition with a line of positive externalities. The narratives of our informants attest to the significant benefit that helping others can have on one’s own well-being—as do decades of research (Thoits and Hewitt, 2001; Musick and Wilson, 2003; Piliavin, 2003; Dolan et al., 2008; Meier and Stutzer, 2008).

Our study also comes with a line of limitations. We have a relatively large sample size for a qualitative study of this type (Marshall et al., 2013; Schreier, 2018), but having only 32 informants who were recruited for sharing their altruistic experience makes their insights primarily representative of certain groups of volunteers in a European context. As researchers embedded in the same sociocultural environment, we must also acknowledge our own reflexive engagement in how we pose questions and interpret answers. The upside to interviewing Slavonic altruists in Norway is that we could compare their experiences to those of Nordic altruists in the same environment. Interviewing Slavonic helpers in their home countries could have engendered different insights, although there is evidence to the effect that the anti-systemic prosociality that has been prevalent among Polish volunteers in Norway has also been the dominant modus operandi in Poland (Helak, 2022). Compared to volunteers in countries nearer Ukraine, such as Poland or Belarus, our informants had had more time to prepare. They were generally not the first helpers to meet the refugees whose experiences with earlier volunteers and reception systems may have influenced their expectations. That relatively few refugees travelled as far north as Norway also affected reception dynamics; the scale of the influx likely impacted the helpers’ intensity of emotion and levels of well-being. Still, applying our MLS model to investigate the role of cultural history and memory in altruistic motivations offers valuable insights.

The mixing of prosocial strategies from formerly authoritarian and liberal-democratic traditions point to prosociality as a learning process with the potential to revitalize cooperation between Western and other populations. Scandocentric universality is part of a Western mode of thought that long was hegemonic, but which in the past decades has lost some of its hold on people’s minds. Challenges from Chinese and Russian powers, Islamist terrorism, and much else have made clear that the world is not on the same path to a Kantian federation of liberal democracies. Instead of being “a city upon a hill,” Americans and many others set examples of political dysfunction and cultural regression. The Nordic nations fare better than many Western countries, but also there, a greater cultural humility has opened people more up to the value of diverse perspectives (Tvedt, 2017). In the narratives of our informants, this attitude expressed itself in an emerging willingness to meet refugees more on their own terms. The Slavonic mode of subversive, inventive altruism proved itself capable also of challenging the hegemony of Nordic thought, similar to the ways in which it previously had undermined communist ideology. Nordic systemic altruism may drive effective policies, but it seems to have less capacity for cross-cultural revitalization. Many Slavonic helpers, however, came to appreciate the efficacy of a voluntarism anchored in social trust and systematic planning.

In our era of ongoing and emerging migration crises, the speed and efficiency of humanitarian help are of paramount importance. Our study emphasizes the need for resilient prosocial strategies that result from cross-cultural translation and learning. We need more comparative research to illuminate how best to care for different groups in flight from famine, war, or political oppression. People with legs in both camps, like our Slavonic informants, seem well suited to revitalize Western modes of humanitarianism. Approaching these processes from an evolutionary perspective can illuminate universal aspects of human altruism. Yet such an approach must be complemented by tools and perspectives developed for studying cultural evolution in particular. Policies that combine an understanding of the idiosyncrasies of our shared human nature with insights into distinct cultural legacies should have a greater chance at success than those informed mostly by the assumptions of one’s own culture.

## Data availability statement

The original contributions presented in the study are included in the article/supplementary material, further inquiries can be directed to the corresponding author.

## Ethics statement

The studies involving human participants were reviewed and approved by the Norwegian Agency for Shared Services in Education and Research (reference number 445357). The participants provided their written or recorded oral informed consent to participate in this study. Written informed consent was obtained from the informants for the publication of any potentially identifiable images or data included in this article.

## Author contributions

ML and NW conceptualized, conducted interviews, and edited the manuscript. ML drafted the manuscript. All authors contributed to the article and approved the submitted version.

## Funding

This work was conducted as part of the multinational, mixed-method Grieg project, which is supported by a European Economic Area grant (project number 2019/34/H/HS6/00597).

## Conflict of interest

The authors declare that the research was conducted in the absence of any commercial or financial relationships that could be construed as a potential conflict of interest.

## Publisher’s note

All claims expressed in this article are solely those of the authors and do not necessarily represent those of their affiliated organizations, or those of the publisher, the editors and the reviewers. Any product that may be evaluated in this article, or claim that may be made by its manufacturer, is not guaranteed or endorsed by the publisher.

## Footnotes

^1^"Slavonic" denotes Eastern European countries with Slavic languages. This is a diverse group in terms of relations to and affinity with Russia. Far from being a monolithic region, these countries practice varying modes of prosociality.

^2^We select these specific nations since they are from where the Nordic and Slavonic interviewees in our study originated.

^3^Oxford Reference defines "prosocial behavior" to be helping, altruistic, or meant to promote the interests of society, https://www.oxfordreference.com/view/10.1093/oi/authority.20110803100350224. Some evolutionary scholars use the term to denote "the social acceptance of all people. Ultimately, Prosocial is an entire worldview," https://www.prosocial.world.

^4^We adumbrate in more detail on this connection in the article "A multilevel selection model for prosocial well-being" (Larsen et al., 2023).

^5^Feeling "good" is not universal, as behaviors motivated by a quest for meaning do not always trigger pleasant emotion. The affect is often described as "feeling right."

^6^For instance, "meaning" was used by several of our informants as synonymous with "purpose." One used "cleaning the floor" as an example of a meaningful activity since it fulfilled a purpose.

^7^Henrich et al. (2010) coined the acronym WEIRD to bring attention to how claims of psychological universals often build on research conducted exclusively on American undergraduates or other Western populations.

## References

[ref1] Andersen L. R. Björkman T. (2017). The Nordic Secret: A European Story of Beauty and Freedom. Stockholm: Fri Tanke.

[ref2] Baucal A. Krstić K. (2020). Searching for an integrative theoretical framework for psychology: evolutionary psychology is needed, but not sufficient. Integr. Psych. Behav. 54, 579–588. doi: 10.1007/s12124-020-09551-2, PMID: 32472482

[ref3] Baumeister R. F. (2005). The Cultural Animal: Human Nature, Meaning, and Social Life. Oxford: Oxford University Press.

[ref4] Baumeister R. F. Vohs K. D. Aaker J. L. Garbinsky E. N. (2013). Some key differences between a happy life and a meaningful life. J. Posit. Psychol. 8, 505–516. doi: 10.1080/17439760.2013.830764

[ref5] Beck E. C. Borevi K. Mouritsen P. (2017). A 'civic turn' in Scandinavian family migration policies? Comparing Denmark, Norway and Sweden. Comp. Migr. Stud. 5:7. doi: 10.1186/s40878-016-0046-7, PMID: 28303235 PMC5331100

[ref6] Bellah R. N. (2011). Religion in Human Evolution: From the Paleolithic to the Axial Age. Cambridge, MA: Harvard University Press.

[ref7] Brandal N. Bratberg Ø. Thorsen D. E. (2013). The Nordic Model of Social Democracy. London: Palgrave Macmillan.

[ref8] Buss D. M. (2000). The evolution of happiness. Am. Psychol. 55, 15–23. doi: 10.1037/0003-066X.55.1.15 11392858

[ref9] Carver C. S. Scheier M. F. (1990). Origins and functions of positive and negative affect: a control-process view. Psychol. Rev. 97, 19–35. doi: 10.1037/0033-295X.97.1.19

[ref10] Conceição P. (2020). The 2020 Human Development Report. United Nations Development Programme.

[ref11] de Puyvallée A. B. Bjørkdahl K. (2021). Do-Gooders at the End of Aid: Scandinavian Humanitarianism in the Twenty-First Century. Cambridge: Cambridge University Press.

[ref12] De Vos M. (2012). “The unbearable lightness of happiness policy” in And the Pursuit of Happiness: Wellbeing and the Role of Government. ed. Booth P. (London: The Institute of Economic Affairs), 181–200.

[ref13] Deaton A. (2010). “Income, aging, health, and well-being around the world: evidence from the Gallup world poll” in Research Findings in the Economics of Aging. Cambridge: National Bureau of Economic Research, 235–263.

[ref14] Diener E. (1984). Subjective well-being. Psychol. Bull. 95, 542–575. doi: 10.1037/0033-2909.95.3.542 6399758

[ref15] Diener E. Oishi S. Lucas R. E. (2015). National accounts of subjective well-being. Am. Psychol. 70, 234–242. doi: 10.1037/a0038899 25844649

[ref16] Dolan P. Peasgood T. White M. (2008). Do we really know what makes us happy? A review of the economic literature on the factors associated with subjective well-being. J. Econ. Psychol. 29, 94–122. doi: 10.1016/j.joep.2007.09.001

[ref17] Eco U. (1986). Semiotics and the Philosophy of Language. Bloomington: Indiana University Press.

[ref18] Finnis J. (2011). Natural Law and Natural Rights. Oxford: Oxford University Press.

[ref19] Fukuyama F. (1992). The End of History and the Last Man. New York: Free Press.

[ref20] Fukuyama F. (2014). Political Order and Political Decay. New York: Farrar, Strauss and Giroux.

[ref21] Gehring K. (2022). Can external threats foster a European unity identity? Evidence from Russia’s invasion of Ukraine. Econ. J. 132, 1489–1516. doi: 10.1093/ej/ueab088

[ref22] Gensicke T. (2000). Freiwilliges engagement in den neuen ländern in Ergebnisse Der Repräsentativerhebung 1999 Zu Ehrenamt, Freiwilligenarbeit Und Bürgerschaftliches Engagement [Schriftenreihe Des Bundesministeriums Für Familie, Senioren, Frauen Und Jugend]. (ed.) von Rosenbladt B. (Stuttgart: Kohlhammer), 176–185

[ref23] Gentile M. (2020). Diabolical suggestions: disinformation and the curious scale of nationalism in Ukrainian geopolitical fault-line cities. Geopolitics Int. Bound. 25, 1–29. doi: 10.1080/14650045.2020.1830766

[ref24] Gentile M. (2022). Pax McDonaldica before the storm: From geopolitical fault-line to urbicide in Mariupol, Ukraine. Trans. Inst. Br. Geogr. doi: 10.1111/tran.12576

[ref25] Harari Y. N. (2014). Sapiens: A Brief History of Humankind. New York: Random House.

[ref26] Heine S. J. (2010). “Cultural psychology” in Handbook of Social Psychology. eds. Fiske S. T. Gilbert D. T. Lindzey G. (Hoboken: John Wiley & Sons), 1423–1464.

[ref27] Helak M. (2022). Polacy wobec Ukraińców: analiza postaw społecznych. Available at: https://www.politykainsight.pl/_resource/multimedium/20314977

[ref28] Helliwell J. F. (2022). World happiness report 2022. Available at: https://worldhappiness.report/ed/2022/

[ref29] Helliwell J. F. Layard R. Sachs J. De Neve J.-E. (2020). World happiness report 2020. Available at: https://worldhappiness.report/ed/2020/

[ref30] Henrich J. (2020). The WEIRDest People in the World: How the West Became Psychologically Peculiar and Particularly Prosperous. New York: Farrar, Straus and Giroux.

[ref31] Henrich J. Heine S. J. Norenzayan A. (2010). The weirdest people in the world? Behav. Brain Sci. 33, 61–83. doi: 10.1017/S0140525X0999152X, PMID: 20550733

[ref32] Hill S. E. Buss D. M. (2008). “Evolution and subjective well-being” in The Science of Subjective Well-Being. eds. Eid M. Larsen R. J. (New York: The Guilford Press), 62–79.

[ref33] Huppert F. A. Marks N. Clark A. Siegrist J. Stutzer A. Vittersø J.. (2009). Measuring well-being across Europe: description of the ESS well-being module and preliminary findings. Soc. Indic. Res. 91, 301–315. doi: 10.1007/s11205-008-9346-0

[ref34] Kakoli R. Ziemek S. (2000). “On the economics of volunteering.” in *ZEF Discussion Papers on Development Policy*, 31, Bonn: University of Bonn, Center for Development Research (ZEF).

[ref35] Kemmelmeier M. Burnstein E. Krumov K. Genkova P. Kanagawa C. Hirshberg M. S.. (2003). Individualism, collectivism, and authoritarianism in seven societies. J. Cross-Cult. Psychol. 34, 304–322. doi: 10.1177/0022022103034003005

[ref36] Kirkpatrick L. A. Navarrete C. D. (2010). Reports of my death anxiety have been greatly exaggerated: a critique of terror management theory from an evolutionary perspective. Psychol. Inq. 17, 288–298. doi: 10.1080/10478400701366969

[ref37] Kornai J. (2021). The Socialist System: The Political Economy of Communism. Princeton: Princeton University Press.

[ref38] Kornai J. Rothstein B. Rose-Ackerman S. (Eds.) (2004). Creating Social Trust in Post-Socialist Transition. London: Palgrave Macmillan.

[ref39] Krastev I. Holmes S. (2020). The Light That Failed: A Reckoning. London: Penguin Books.

[ref40] Krys K. Capaldi C. A. Zelenski J. M. Park J. Nader M. Kocimska-Zych A.. (2021a). Family well-being is valued more than personal well-being: a four-country study. Curr. Psychol. 40, 3332–3343. doi: 10.1007/s12144-019-00249-2

[ref41] Krys K. Park J. Kocimska-Zych A. Kosiarczyk A. Selim H. A. Wojtczuk-Turek A.. (2021b). Personal life satisfaction as a measure of societal happiness is an individualistic presumption: evidence from fifty countries. J. Happiness Stud. 22, 2197–2214. doi: 10.1007/s10902-020-00311-y

[ref42] Kuti E. (2004). Civic service in Eastern Europe and Central Asia: from mandatory public work toward civic service. Nonprofit Volunt. Sect. Q. 33, 79S–97S. doi: 10.1177/0899764004269740

[ref43] Larsen M. (2021). The Lutheran imaginary that underpins social democracy. Front. Psychol. 12:746406. doi: 10.3389/fpsyg.2021.746406, PMID: 34566825 PMC8460851

[ref44] Larsen M. (2022). Bridging the Narrative Abyss: Evolution Toward Social Democracy in a Millennium of Nordic Fiction. PhD. University of California, Los Angeles.

[ref45] Larsen M. Witoszek N. Yeung J. C. (2023). A multilevel selection model for prosocial well-being. Front. Psychol. 14:1068119. doi: 10.3389/fpsyg.2023.1068119 36910840 PMC9995435

[ref46] Levitt H. M. Motulsky S. L. Wertz F. J. Morrow S. L. Ponterotto J. G. (2017). Recommendations for designing and reviewing qualitative research in psychology: promoting methodological integrity. Qual. Psychol. 4, 2–22. doi: 10.1037/qup0000082

[ref47] Lewis D. M. G. Al-Shawaf L. Russell E. M. Buss D. M. (2015). “Friends and happiness: an evolutionary perspective on friendship” in Friendship and Happiness. ed. Demir M. (Berlin: Springer), 37–57.

[ref48] Lotman J. (1990). The Universe of the Mind. Kolkata: Taurus.

[ref49] Lumsden C. J. Wilson E. O. (2005). Genes, Mind, and Culture: The Coevolutionary Process. Cambridge, MA: Harvard University Press.

[ref50] Marshall B. Cardon P. Poddar A. Fontenot R. (2013). Does sample size matter in qualitative research? a review of qualitative interviews in is research. J. Comput. Inf. Syst. 54, 11–22. doi: 10.1080/08874417.2013.11645667

[ref51] Meier S. Stutzer A. (2008). Is volunteering rewarding in itself? Economica 75, 39–59. doi: 10.1111/j.1468-0335.2007.00597.x

[ref52] Meyer T. (2007). The theory of social democracy. Cambridge: Polity

[ref53] Musick M. A. Wilson J. (2003). Volunteering and depression: the role of psychological and social resources in different age groups. Soc. Sci. Med. 56, 259–269. doi: 10.1016/S0277-9536(02)00025-4, PMID: 12473312

[ref54] Nelson R. (2017). Lutheranism and the Nordic Spirit of Social Democracy: A Different Protestant Ethic. Aarhus: Aarhus University Press.

[ref55] Nesse R. M. (2005). “Natural selection and the elusiveness of happiness” in The Science of Well-Being (Online). eds. Huppert F. A. Baylis N. Keverne B. (Oxford: Oxford University Press)

[ref56] Norman K. (2018). Sweden's Dark Soul: The Unravelling of a Utopia C. London: Hurst & Co.

[ref57] Piliavin J. A. (2003). “Doing well by doing good: benefits for the benefactor” in Flourishing: Positive Psychology and the Life Well-Lived. eds. Keyes C. L. M. Haidt J. (Washington, DC: American Psychological Association), 227–247.

[ref58] Plagnol A. C. Huppert F. A. (2010). Happy to help? Exploring the factors associated with variations in rates of volunteering across Europe. Soc. Indic. Res. 97, 157–176. doi: 10.1007/s11205-009-9494-x

[ref59] Rappleye J. Komatsu H. Uchida Y. Krys K. Markus H. (2020). ‘Better policies for better lives’?: constructive critique of the OECD’s (mis)measure of student well-being. J. Edu. Policy 35, 258–282. doi: 10.1080/02680939.2019.1576923

[ref60] Ricard M. (2015). Altruism. Boston: Little Brown and Company.

[ref61] Rothstein B. (2005). Social Traps and the Problem of Trust. Cambridge: Cambridge University Press.

[ref62] Røysamb E. Nes R. B. (2016). “Genes, environments, and core features of eudaimonic wellbeing” in Handbook of Eudaimonic Well-Being. ed. Vittersø J. (London: Springer), 233–252.

[ref63] Schreier M. (2018). “Sampling and generalization” in The SAGE Handbook of Qualitative Data Collection. ed. Flick U. (London: SAGE Publications), 84–98.

[ref64] Skagen K. (2018). Norge, Vårt Norge: Et Lands Biografi. Oslo: Dreyers forlag.

[ref65] Solomon S. Greenberg J. Pyszczynski T. (2000). Pride and prejudice: fear of death and social behavior. Curr. Dir. Psychol. Sci. 9, 200–204. doi: 10.1111/1467-8721.00094

[ref66] Thoits P. A. Hewitt L. N. (2001). Volunteer work and well-being. J. Health Soc. Behav. 42, 115–131. doi: 10.2307/3090173 11467248

[ref67] Trägårdh L. (2013). “The historical incubators of trust in Sweden: from the rule of blood to the rule of law” in Trust and Organizations. eds. Reuter M. Wijkstrom F. Uggla B. K. (London: Palgrave Macmillan), 181–203.

[ref68] Tvedt T. (2017). Det Internasjonale Gjennombruddet: Fra “Ettpartistat” Til Flerkulturell Stat. Oslo: Dreyers forlag.

[ref69] Uchida Y. Kitayama S. (2009). Happiness and unhappiness in east and west: themes and variations. Emotion 9, 441–456. doi: 10.1037/a0015634, PMID: 19653765

[ref70] Uchida Y. Townsend S. S. M. Markus H. R. Bergsieker H. B. (2009). Emotions as within or between people? Cultural variation in lay theories of emotion expression and inference. PSPB 35, 1427–1439. doi: 10.1177/0146167209347322 19745200

[ref71] UDI (2023). Statistikk om Ukrainasituasjonen. Available at: https://www.udi.no/statistikk-og-analyse/ukraina/

[ref72] UNHCR (2023). Operational data portal: Ukraine refugee situation. Available at: https://data.unhcr.org/en/situations/ukraine.

[ref73] Vittersø J. (n.d.). Oh, my goodness: Towards a humanistic theory of wellbeing.

[ref74] Welzel C. (2013). Freedom Rising: Human Empowerment and the Quest for Emancipation. Cambridge: Cambridge University Press.

[ref75] Wilson D. S. Coan J. A. (2021). Groups as organisms: implications for therapy and training. Clin. Psychol. Rev. 85:101987. doi: 10.1016/j.cpr.2021.101987 33725511

[ref76] Wilson D. S. Hayes S. C. Biglan A. Embry D. D. (2014). Evolving the future: toward a science of intentional change. Behav. Brain Sci. 37, 395–416. doi: 10.1017/S0140525X13001593, PMID: 24826907 PMC4331065

[ref77] Wilson D. S. Hessen D. O. (2018). “Cooperation, competition, and multi-level selection: a new paradigm for understanding the Nordic model” in Sustainable Modernity: The Nordic Model and Beyond. eds. Witoszek N. Midttun A. (London: Routledge), 18–35.

[ref78] Witoszek N. (2007). “Friendship and revolution in Poland: the eros and ethos of the committee for workers' defense (KOR)” in Friendship in Politics: Theorizing Amity in and Between States (eBook). eds. King P. Smith G. M., vol. 10 (London: Routledge), 215–231.

[ref79] Witoszek N. (2011). The Origins of the “Regime of Goodness”: Remapping the Cultural History of Norway. Oslo: Universitetsforlaget.

[ref80] Witoszek N. (2019). “The profits and pitfalls of prosociality: cultural-evolutionary perspectives on Scandinavia” in The Relational Nordic Welfare State: Between Utopia and Ideology. eds. Hänninen S. Lehtelä K.-M. Saikkonen P. (Northampton: Edward Elgar), 50–72.

[ref81] Witoszek N. Midttun A. (Eds.) (2018). Sustainable Modernity: The Nordic Model and Beyond. London: Routledge.

[ref82] WVS (2020). Most people can be trusted. Available at: https://www.worldvaluessurvey.org/WVSOnline.jsp

[ref83] Zagaria A. Ando A. Zennaro A. (2020). Psychology: a giant with feet of clay. Integr. Psych. Behav. 54, 521–562. doi: 10.1007/s12124-020-09524-5, PMID: 32297037

[ref84] Zagaria A. Ando A. Zennaro A. (2021). Toward a cultural evolutionary psychology: why the evolutionary approach does not imply reductionism or determinism. Integr. Psychol. Behav. Sci. 55, 225–249. doi: 10.1007/s12124-021-09613-z 33880709

