# Corrigendum: Strategies of prosociality: comparing Nordic and Slavonic altruism toward Ukrainian refugees

**DOI:** 10.3389/fpsyg.2023.1264744

**Published:** 2023-08-01

**Authors:** Mads Larsen, Nina Witoszek

**Affiliations:** Centre for Development and the Environment, University of Oslo, Oslo, Norway

**Keywords:** anti-systemic altruism, authoritarianism, evolutionary psychology, migration, multilevel selection, qualitative interviews, systemic altruism, well-being

In the published article, there was an error in the Funding statement. The statement previously said: “This work was conducted as part of the multinational, mixed-method “Grieg” project, which is supported by a European Economic Area grant (award number 103194100).” The corrected Funding statement appears below.

